# Androgen Receptor-Positive Penile Acrochordon: A Case Report

**DOI:** 10.7759/cureus.58177

**Published:** 2024-04-13

**Authors:** Serkan Akan, Umut Arslan, Esin Yiğitbaşi Kiliç

**Affiliations:** 1 Department of Urology, University of Health Sciences Fatih Sultan Mehmet Training and Research Hospital, Istanbul, TUR; 2 Department of Pathology, University of Health Sciences Fatih Sultan Mehmet Training and Research Hospital, Istanbul, TUR

**Keywords:** fibroepithelial polyp, skin tag, penile acrochordon, androgen receptor, acrochordon

## Abstract

Acrochordons are polypoid, skin-colored lesions usually present at the site of skin folds. They are extremely rare in the preputial area of the penis and do not tend to grow. To the best of our knowledge, in English literature, this report presents the first case of an androgen receptor-positive penile acrochordon, which is located on the penis and showed rapid growth along with body development during puberty with no underlying causes such as acromegaly, diabetes, obesity, and trauma.

## Introduction

Acrochordons, also known as “fibroepithelial polyp” or “skin tag,” are insignificant, benign, common skin lesions containing varying amounts of stroma, surrounded by the histologically papillomatous epidermis. They are typically asymptomatic and found in the cervical, axillary, and inguinal regions [1]. They may be associated with diabetes, obesity, hyperlipidemia, insulin resistance, acromegaly, and trauma [2]. Physical examination is usually sufficient for diagnosis, but in rare cases, histopathological examination may be necessary due to an unusual site of presentation and no underlying causes [3]. To the best of our knowledge, in English literature, we report the first case of an androgen receptor-positive penile acrochordon located on the glans penis, which showed rapid growth along with body development during puberty.

## Case presentation

**Figure 1:**
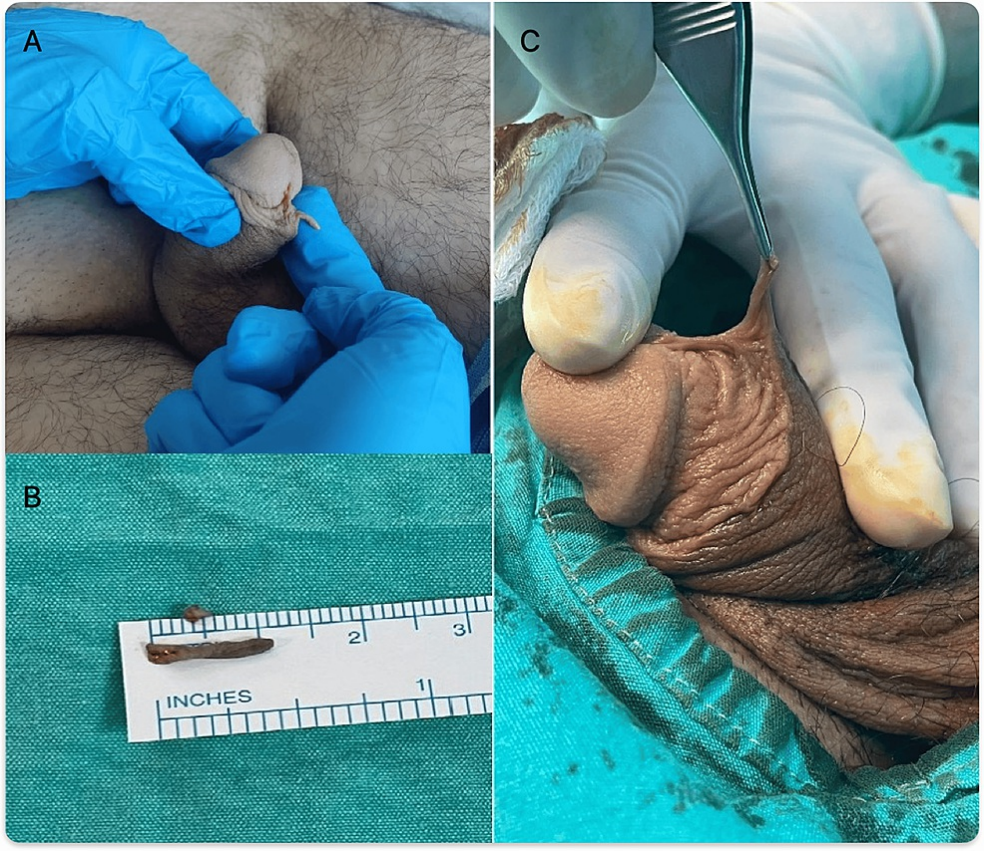
Penile acrochordon. (A) Physical examination before surgery, (B) specimen of acrochordon, and (C) intraoperative examination of acrochordon.

A 16-year-old adolescent male presented with an asymptomatic, horn-like, skin-colored lesion on the preputial mucosa of the penis. About three years ago, a 1-2 mm skin tag was observed, which progressively grew after puberty. He had not engaged in sexual intercourse yet and had no history of genital herpes or genital warts. He had no skin moles elsewhere on his body and reported no urinary tract infections or voiding symptoms. He underwent circumcision surgery at the age of 11. His physical examination revealed a skin lesion just over 1 cm in length on the ventral side of the penis. It was located on the circumcision line and was in close proximity to the frenulum but was not associated with the urethra (Figure 1).

**Figure 2:**
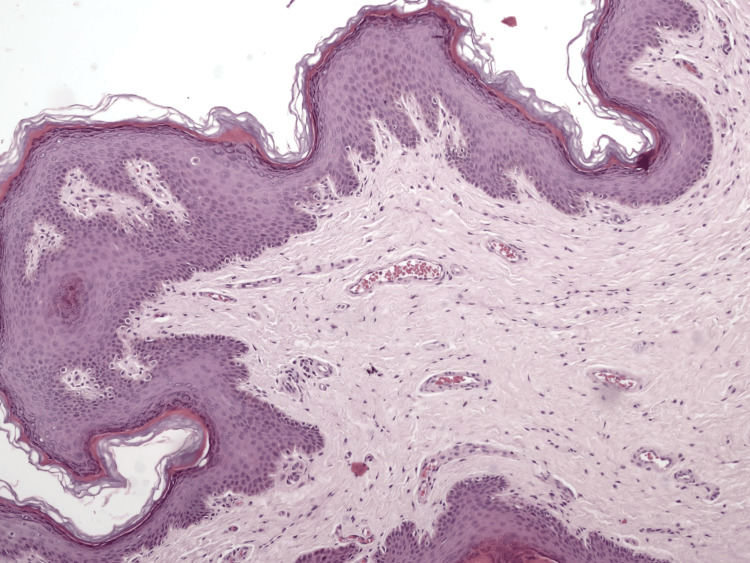
Polypous lesion lined with keratinized multilayered squamous epithelium with surface indentations (H&E x100).

His inguinal and scrotal examination was normal. Routine blood tests (CBC, basic metabolic panel (including blood glucose), lipid panel, hormonal panel, and HbA1C) were within normal limits, VDRL was non-reactive, and HIV 1 and 2 tests were negative. The lesion was excised under general anesthesia as per the patient's request and sent for histopathological examination. The sections revealed a polypous lesion lined with keratinized multilayered squamous epithelium with surface indentations. The multilayered squamous epithelium was acanthotic in some areas, but there were no signs of atypia. The subepithelial stroma was edematous and slightly cellular and contained fibroblastic cells with elongated oval nuclei with indistinct cytoplasmic borders (Figure 2). 

**Figure 3:**
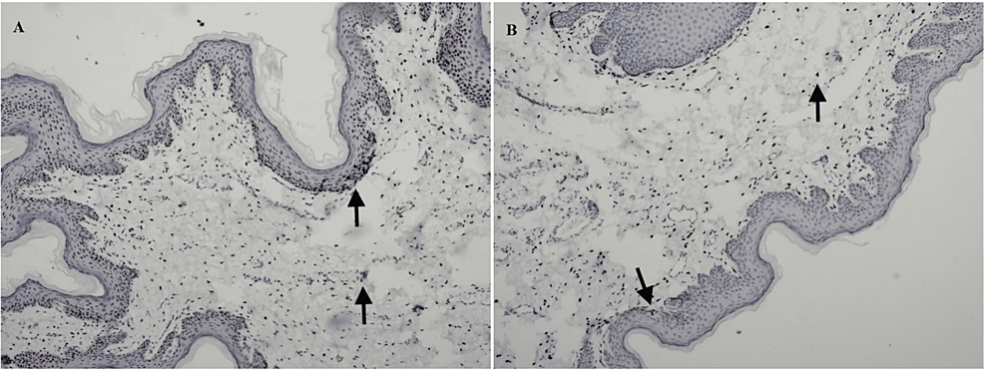
Immunohistochemical staining (IHC x100). (A) Staining for androgen receptors: Intranuclear staining in stratified squamous epithelium and some stromal cells. (B) Staining for progesterone receptors: Intranuclear staining in stratified squamous epithelium and some stromal cells.

Hormonal receptor density was examined by immunohistochemical staining of the sections. Since the lesion grows during adolescence and is in a rare location, staining was performed with androgen, progesterone, estrogen, and human papilloma virus (HPV). Both squamous epithelial and stromal fibroblastic cells showed intense staining for androgen receptors (Figure 3A). Progesterone receptors were negative in squamous epithelium but were seen in stromal fibroblastic cells (Figure 3B). 

No staining for estrogen receptors was observed. HPV types 6, 16, 18, 31, 51, 52, 53, 56, 58, and 66 were negative. In the sixth postoperative month, his physical examination was normal, and no recurrence was observed.

## Discussion

Acrochordons are benign, small skin lesions that occur in the skin folds such as neck, axilla, and groin [1]. Skin tags can occur at any age. They are typically asymptomatic, usually smaller than 5 mm in length, and rarely exceed 1 cm [3]. Microscopically, an acrochordon consists of a fibrovascular core, sometimes also with fat cells, covered by an unremarkable epidermis or an acanthotic, flattened, or frond-like epithelium [4].

The prevalence of acrochordons in the general population is approximately 46% [4]. They generally neither grow nor change over time. Genetic factors and trauma were postulated for their etiology due to the preferred areas on the skin and familial predisposition. Previous studies have demonstrated the role of HPV 6 and 11 in the pathogenesis of genital acrochordons [4]. Skin tags may be an important marker for the presence of type II diabetes mellitus [5]. The incidence is increased in patients with obesity, hyperlipidemia, insulin resistance, and acromegaly; therefore, hormonal factors are also considered [3]. However, unlike the literature, in our case, the lesion grew rapidly along with body development during puberty and reached a size approximately 5-10 times larger than the initial size. Although the literature suggests a steady size over time, we observed a definite growth during puberty in our case. Therefore, we analyzed the hormonal receptor density by immunohistochemical examination. The intense positivity of androgen receptors suggested that hormonal factors may play a role in the development of acrochordons.

The relationship between the hormonal receptors and the development of the genital tubercles in fetal mice is discussed in the literature. Some studies demonstrated that progesterone receptors played a significant role in the development of genital tubercles [6]. Exposure to certain doses of estrogen during fetal development in males can result in a shift towards the female end of the spectrum, leading to hypospadias [7]. Androgens, which increase during puberty, may affect the growth of skin tags due to their anabolic effects. Previous studies demonstrated the anabolizing effects of insulin-like growth factor 1 receptor (IGF-1R) and IGF-2R on acrochordon formation, but there is no study showing similar effects of the androgens [8]. Further research is required to investigate the association of androgen and progesterone exposure and receptor upregulation in the tissue with the formation and growth of the skin lesions. 

Skin tags are benign, and treatment is usually unnecessary unless the tags are irritating or cosmetically problematic. If removal of an acrochordon is desired or warranted, cauterization, cryosurgery, surgical ligation, or excision may be used [8].

## Conclusions

To the best of our knowledge, in English literature, we report the first case of an androgen receptor-positive penile acrochordon located on the glans penis, which showed rapid growth along with body development during puberty. Further research is required to investigate the association of androgen and progesterone exposure and receptor upregulation in the tissue with the formation and growth of the skin lesions.
